# Carotid artery stenting and endarterectomy surgery techniques: A 30‑year time‑lapse

**DOI:** 10.3892/mi.2023.121

**Published:** 2023-11-16

**Authors:** Petroula Nana, Konstantinos Spanos, George Kouvelos, Vasiliki Epameinondas Georgakopoulou, Ioannis G. Lempesis, Nikolaos Trakas, Pagona Sklapani, Konstantinos Paterakis, George Fotakopoulos, Alexandros G. Brotis

**Affiliations:** 1 Department of Vascular Surgery, Larissa University Hospital, Faculty of Medicine, School of Health Sciences, University of Thessaly, 41221 Larissa, Greece; 2 Department of Pathophysiology, National and Kapodistrian University of Athens, 11527 Athens, Greece; 3 Department of Biochemistry, Sismanogleio Hospital, 15126 Athens, Greece; 4 Department of Neurosurgery, General University Hospital of Larissa, 41221 Larissa, Greece

**Keywords:** carotid artery stenting, carotid stenosis, angioplasty, bibliometrics, statistics

## Abstract

Carotid endarterectomy or carotid artery stenting (CAS), are the most important axes in carotid artery interventional management. A bibliometric analysis permits an easier access to the current literature trends and information to design future studies. The aim of the present study was to identify the knowledge routes on CAS and examine the research front on the topic. The search was interpreted in Scopus, from 1994 to 2023, and included only original articles and reviews. The BibTex format was used to download all citation and bibliographic data. The present analysis was conducted in two parts, a descriptive one and a network extraction process. Between 1994 and 2023, 34,503 references and 7,758 authors were recorded. The annual growth rate was 21.64%. The CAVATAS trial was the most cited article. As regards word trends, since 2017, trans-carotid stenting, risk factors and plaque characteristics are highlighted. CAS remains an area of high interest with a publication growth rate of >20% per year. As numerous questions remain to be answered, the need to determine the role of CAS may drive further research.

## Introduction

**Figure 1:**
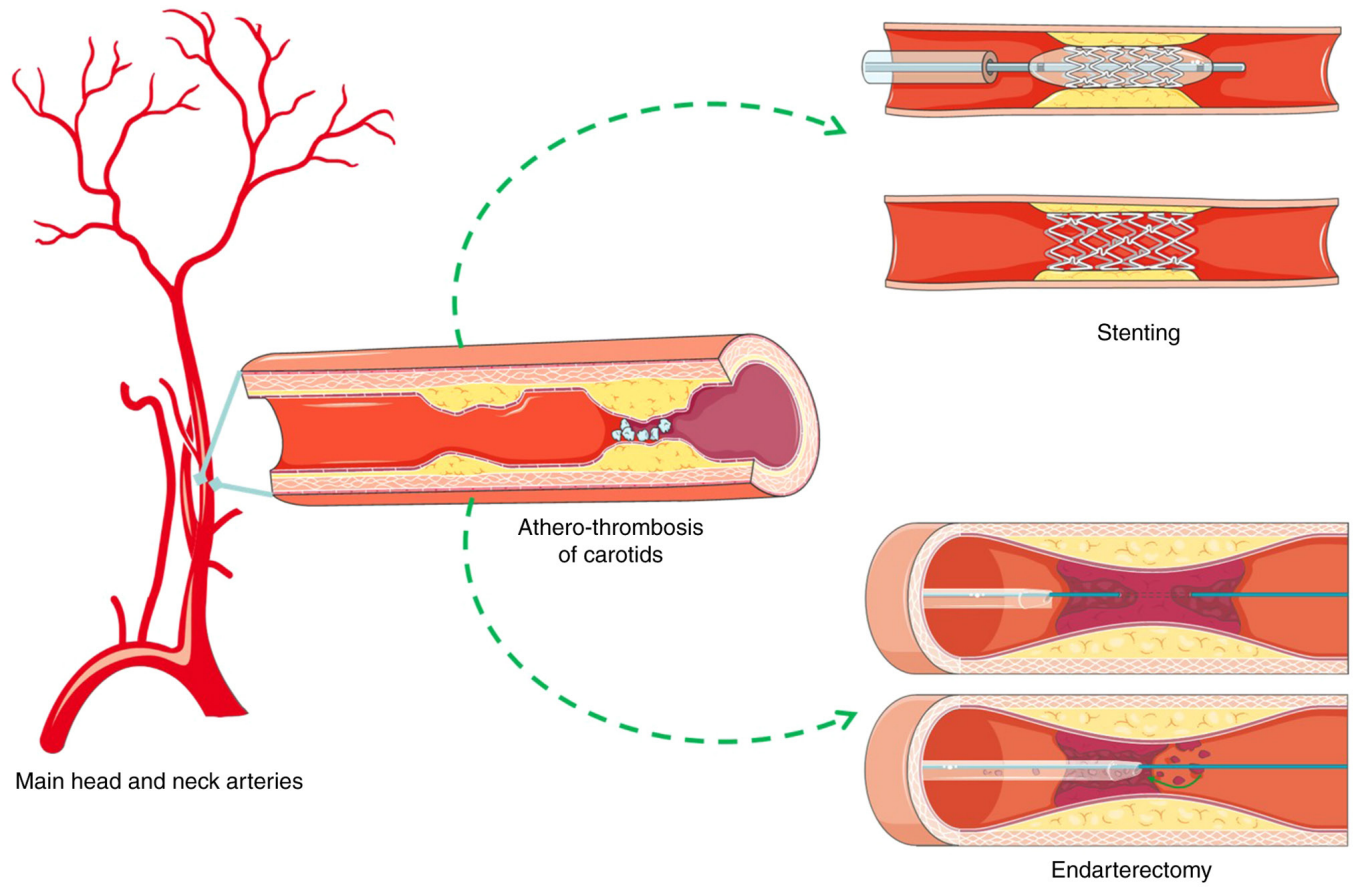
Schematic illustration of the main head and neck arteries, carotid athero-thrombosis and endarterectomy and stenting procedures. Parts of this image were derived from the free medical site, http://smart.servier.com/ (accessed on July 18, 2023) by Servier, licensed under a Creative Commons Attribution 3.0 Unported License.

Stroke remains main causative factor of mortality and disability worldwide (1,2). The aging of the population at >65 years is positively associated with the crude mortality and incidence of stroke, according to the World Health Organization, while western countries are among the regions with the highest mortality rates due to stroke (3-5). Carotid artery disease, a subcategory of peripheral artery disease, is associated with the incidence of stroke (1,6-8). Medical treatment and interventional management with carotid endarterectomy or carotid artery stenting (CAS) are the main axes in the management of carotid artery disease (Fig. 1) (1,6). However, to the best of our knowledge, no randomized trial comparing the interventional approaches has been conducted to date to prove the superiority of CAS, while studies are under way to shed some light on its application and outcomes (9-11).

In the latest guidelines, researchers have noted the need for presenting newer evidence regarding the management of carotid stenosis, the prevention of stroke, the timing of interventions, as well as novelties regarding endovascular techniques (1,6,12,13). A bibliometric analysis would permit the assessment of the currently available literature and reflect the synchronous trends in a number of different aspects, such as carotid stenting, which remains an evolutionary field. Such an analysis depicts the association between institutions and authors, and estimates the publication rates among countries and journals. Along this line, vascular specialists may have easier access to current literature trends and information to design future studies.

The primary aim of the present study was to identify the knowledge routes in CAS using bibliometrics, a systematic and reproducible statistical analysis (9). The secondary aims were to examine the research front on the topic under study and produce a social network of the associated scientific community.

## Data and methods

### Study workflow

The workflow recommended for science mapping, using the statistical environment R and Biblioshiny interphase (package bibliometrix), was used to conduct the bibliometric analysis (14). In the present study, Bibliometrix, a popular tool for bibliometric analysis, was used (14).

### Search strategy

An electronic search in Scopus, focusing on articles reporting on CAS, was performed using ‘carotid artery surgery’ OR ‘carotid endarterectomy’ AND ‘endovascular’ OR ‘carotid artery stenting’ as the search terms. The search was performed from 1994 to 2023, and included only original studies and reviews written in the English language. Conference articles, letters to the editor, editorials, books, notes, short surveys and errata were excluded from the current analysis.

### Data collection

The BibTex format was used to download all citation and bibliographical data, including the abstracts and key words of the eligible records. The article title, authors, journal, year of publication, Scopus citations, citation count, number of authors, first and senior author names, and the country of the corresponding author were used for the bibliometric analysis. All data, without any further filtering, were loaded and analyzed on Biblioshiny.

### Analysis of the obtained data

The present study was conducted in two parts, a descriptive one and a network extraction process. The descriptive analysis retrieved evidence on the most productive authors and countries, the most cited articles, the most frequent journals, and the most common key words of authors, and the results are presented in tables. In the network extraction process, three sub-analyses were performed, including a collaboration analysis, according to institutions and countries, a co-citation analysis based on authors, and a word co-occurrence analysis according to the key words of authors.

### Data visualization

Trends and temporal data were visualized using burst detection and simple time-series plots. The word co-occurrence, institution collaboration, and author co-citation analyses were presented in word proximity maps. Geospatial data are presented in geographical maps, while conceptual structures are presented in cluster strings.

## Results

### Results of the literature search

**Figure 2:**
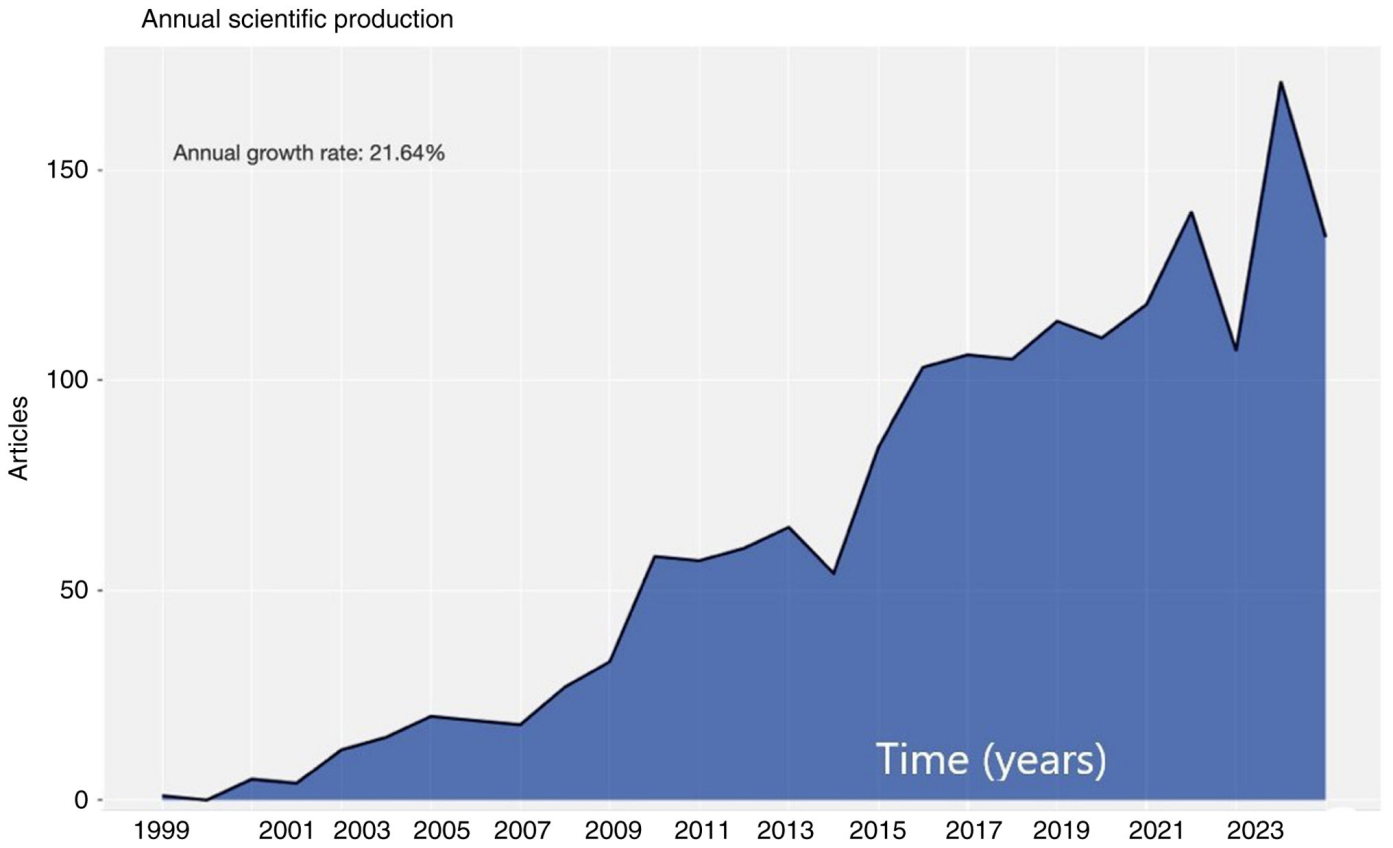
The increasing interest in carotid artery stenting is depicted in a literature annual growth rate estimated at 21.64%.

The search on Scopus resulted in 2,604 records. A total of 225 records were excluded due to language restrictions. An additional 405 articles were discarded for inappropriate study design. Finally, 1,974 articles were included in the present analysis (Fig. S1). Between 1994 and 2023, 34,503 references and 7,758 authors were recorded. The average citation rate per document was 16.67 in the total time span (1,792 average citations per year/document) while 0.224 documents per author were recorded. The annual growth rate was 21.64%, reflecting the increasing interest in CAS (Fig. 2).

### Top-20 most cited documents, journals and productive authors

The list of the top 20 most cited articles is presented in Table I. The CAVATAS trial (15) was the most cited article among the original articles, followed by the European Society of Cardiology Guidelines regarding the management of carotid stenosis published in 2011 and the Global Experience on Carotid Artery Stenting published by Wholey *et al* (16). The USA was the most frequent country of the corresponding author, while American universities were the most implicated in CAS publications.

### The top 20 most common keywords and word trends

**Figure 3:**
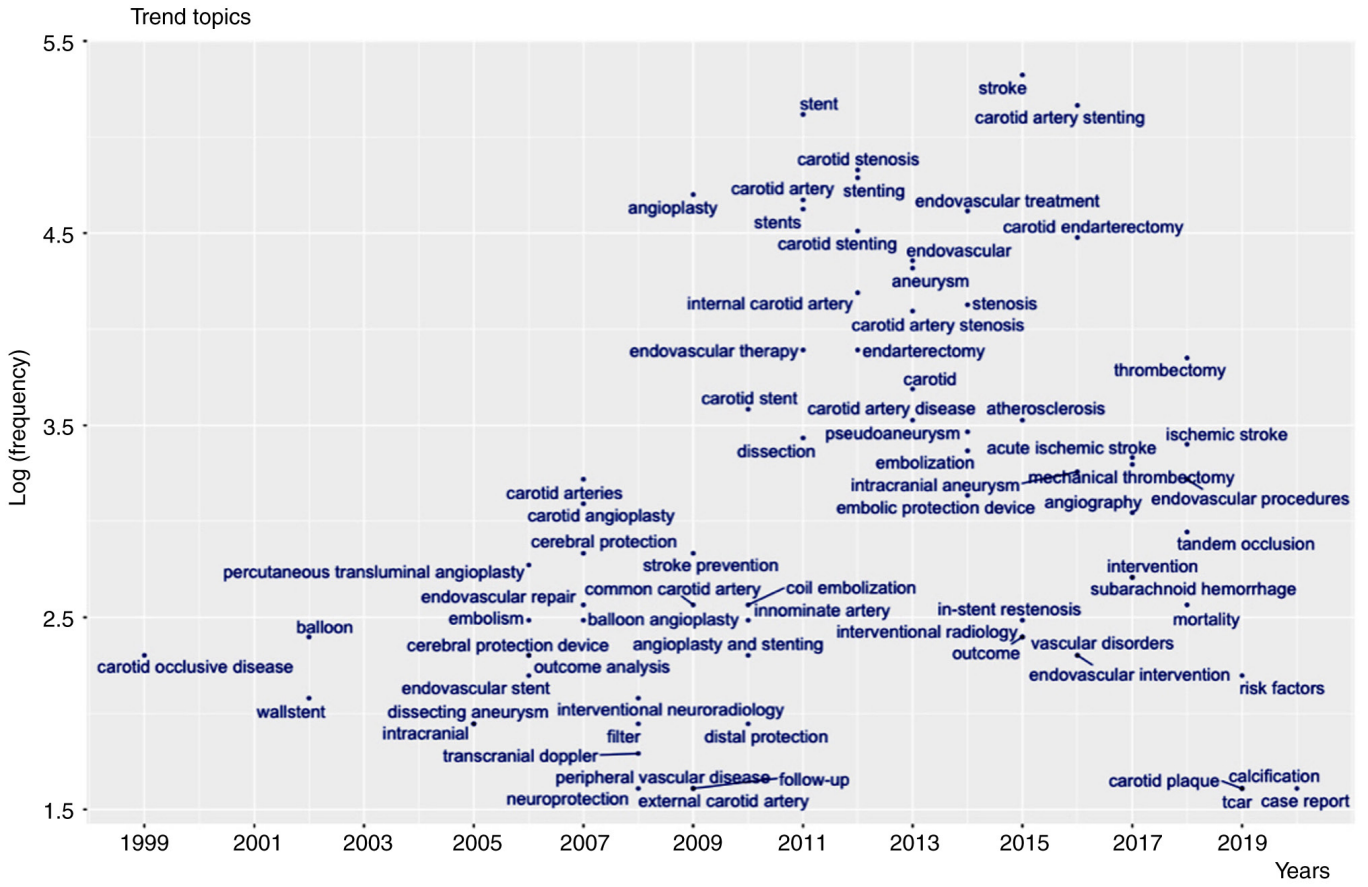
Trends regarding word growth. Regarding word trends, since 2017, trans-carotid stenting, risk factors and plaque characteristics are highlighted.

The common key words and trends of authors regarding their word growth are presented in Table II and Fig. 3. Stroke, CAS and stent were the most commonly used key words. As regards word trends, since 2017, trans-carotid stenting (TCAR), risk factors and plaque characteristics have been highlighted.

### Collaboration analysis

**Figure 4:**
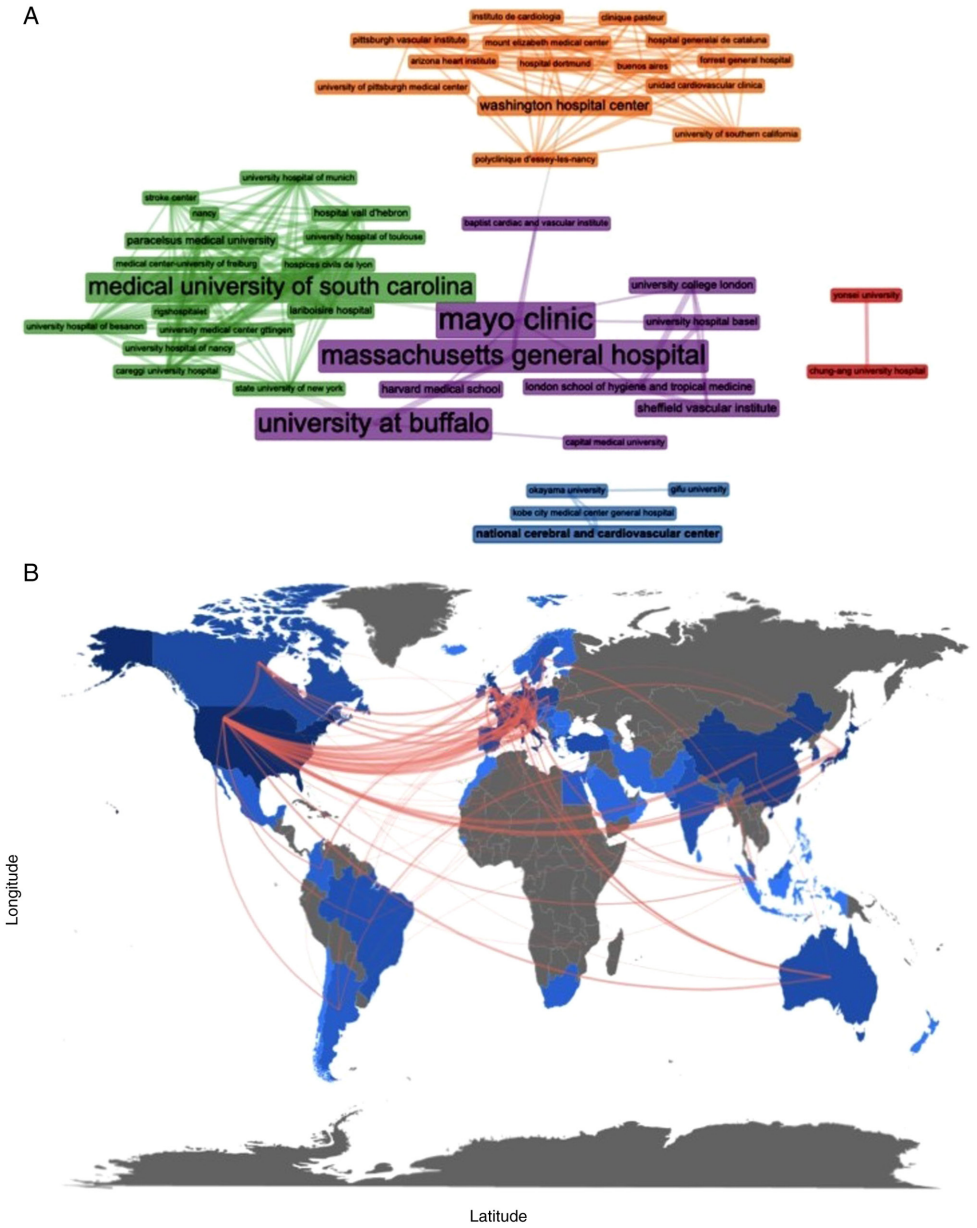
(A) A total of 20 institutions were implicated in publications on carotid artery stenting. Institutions from the USA, as well as Japanese universities created three cores. (B) Collaborations were observed between European countries and the USA.

In terms of the collaboration analysis, 20 institutions were implicated in CAS publications, with three important clusters (Fig. 4A). Institutions from the USA, as well as Japanese universities created three cores, while other collaborations were observed between European countries and the USA (Fig. 4B).

## Discussion

In this analysis, CAS literature progression was summarized over a 25-year period. CAS remains a critical issue in vascular surgery. The application of minimal and novel interventions, such as TCAR, and technology evolution regarding stents and embolic protection devices, such as reversed flow, will probably affect the application of CAS in the future. Current recommendations have not established the role of CAS in carotid stenting management (1,6). At 10 years after the initial studies, randomized trials continue to evolve regarding the role of CAS in carotid stenosis management (17). The annual growth rate of CAS literature is >20%, indicating current interest. Over the past few years, analogous bibliometric analyses have been performed regarding carotid artery stenosis, reflecting the interest of the vascular and other scientific communities in this field (18-20).

The top three studies include the initial randomized trial in CAS, the guidelines of the European Society of Cardiology, and a multicenter study. In 2001, the first multicenter randomized trial, the CAVATAS study, presented the comparative results of angioplasty versus endarterectomy in patients with carotid and vertebral stenosis (1,247 citations) (15). The authors concluded that carotid or vertebral angioplasty presented similar major risks and effectiveness in stroke prevention during the initial 3 years of follow-up (21). The European Society of Cardiology published in 2011 recommendations regarding the management of atherosclerotic carotid and vertebral, mesenteric, renal, upper, and lower extremity artery disease (1,145 citations) (16). The revision of these recommendations was published in 2017 with the collaboration of the European Society of Cardiology and Vascular Surgery (6). CAS recommendations present the same level of evidence (level of evidence IIb) (6,21). However, the ‘high volume center’ criterion was replaced by plaque characteristics and life expectancy (6,21). Carotid plaque is among the word trends, indicating the focus of research on the role of the plaque in stroke risk stratification and indications for surgical treatment. The top three studies also include the multicenter study of Wholey *et al* (16), published in 2000. The purpose of that article was to review CAS placement worldwide using a survey of multiple questions regarding patient selection, techniques, and CAS outcomes (16). In total, 5,210 procedures and 4,757 patients were included in that analysis. Early experience in CAS demonstrated highly acceptable results in terms of minor and major cerebral events and mortality (16).

Vascular surgery, cardiovascular disease and neuro-interventional journals were the hosts of all records. Stroke, CAS and stent were the most commonly used key words, followed by carotid stenosis and angioplasty. The word growth and trend topics based on the key words of authors revealed that CAS is an evolutionary issue with an increased impact in vascular surgery literature. Notably, an increasing interest has risen regarding the application of TCAR, which is associated with a low morbidity and mortality rate (22-24). As endovascular management gains popularity among specialists from different scientific fields, it appears that CAS literature will probably serve future studies.

In terms of collaboration analysis, an extensive network among 20 institutions is provided regarding CAS publications. A total of three large collaboration networks were identified, including universities from the USA and two smaller ones represented by Japanese institutions. Other important collaborations were observed between European countries, such as the United Kingdom, France, Germany and the USA. Collaboration among Universities, specialists and countries is the cornerstone of medical evolution, as well as vascular surgery. Multidisciplinary approaches gain popularity, facilitate patient management, ameliorate outcomes and reduce complications in other fields of vascular medicine (25,26). The role of the multidisciplinary approach is further emphasized in the available recommendations (1,6).

The present study has certain limitations, which should be mentioned. The current analysis provides a holistic view of the scientific research of CAS from 1994 to 2023. The inclusion of most articles and reviews limited the inherent bias of this analysis. However, missing significant publications cannot be precluded, as time for citation accumulation may be required. As only corresponding authors were included in the analysis, the performance of other coauthors could not be analyzed.

In conclusion, CAS remains an area of high interest among specialties, with a publication growth rate estimated at >20% per year. As numerous questions remain to be answered, the need to determine the role of CAS may drive further research and, subsequently, publications.

## Supplementary Material

Data extraction flow of the present study. The final analysis included 1,974 articles.

## Figures and Tables

**Table I:** Data synthesis of the included studies.

Author(s), year of publication	Country and University	Study design	Total citations per year	(Refs.)
Tendera *et al*, 2011	Poland, Medical University of Silesia	Review	114.5	(21)
Brown *et al*, 2001	UK, University College London	Retrospective	62.35	(15)
Wholey *et al*, 2000	USA, University of Texas Health Science Center at San Antonio	Prospective	23.1	(16)
Biasi *et al*, 2004	Italy, University of Milan-Bicocca	Review	22.7	(27)
Alonso-Coello *et al*, 2012	Multicenter	Review	20.77	(28)
Cremonesi *et al*, 2003	Italy	Prospective	17.5	(29)
Cronenwett *et al*, 2012	USA	Prospective	17.22	(30)
Schnaudigel *et al*, 2008	Germany, University of Göttingen	Review	16.69	(31)
Bonati *et al*, 2009	UK, University College London	Prospective	15.41	(32)
Ederle *et al*, 2009	UK, University College London		14.41	(33)
Ansel *et al*, 2010	USA	Prospective	14.00	(34)
Wholey *et al*, 1998	USA, Louisiana State University Medical Center	Review	10.74	(35)
Ohki *et al*, 1998	USA, The University Hospital for the Albert Einstein College of Medicine	Prospective	9.65	(36)
Meyers *et al*, 2000	USA, University of California at San Francisco	Retrospective	8.90	(37)
Grabenwöger *et al*, 2000	Austria, University of Vienna	Prospective	7.47	(38)
Sullivan *et al*, 1998	USA	Prospective	7.21	(39)
Lai and Hobson, 2000	USA, University of Medicine and Dentistry-New Jersey Medical School	Prospective	7.09	(40)
Wholey *et al*, 1997	USA	Prospective	6.91	(41)

**Table II:** Characteristics of the studies.

Words	Occurrences
Stroke	205
Carotid artery stenting	175
Stent	167
Carotid stenosis	125
Stenting	120
Angioplasty	110
Carotid artery	107
Stents	102
Endovascular treatment	101
Carotid stenting	91
Carotid endarterectomy	88
Endovascular	78
Aneurysm	75
Internal carotid artery stenosis	66
Stenosis	62
Carotid artery stenosis	60
Endarterectomy	49
Endovascular therapy	49
Thrombectomy	47
Carotid	40

## Data Availability

The datasets used and/or analyzed during the current study are available from the corresponding author on reasonable request.
