# Supplementary figures and images for: Carotid artery stenting and endarterectomy surgery techniques: A 30‑year time‑lapse
Source: Med Int (Lond). 2023 Nov 16;3(6):61. doi: 10.3892/mi.2023.121 (PMC10777268; doi:10.3892/mi.2023.121)

Figure S1. Data extraction flow of the present study. The final analysis included 1,974 articles.

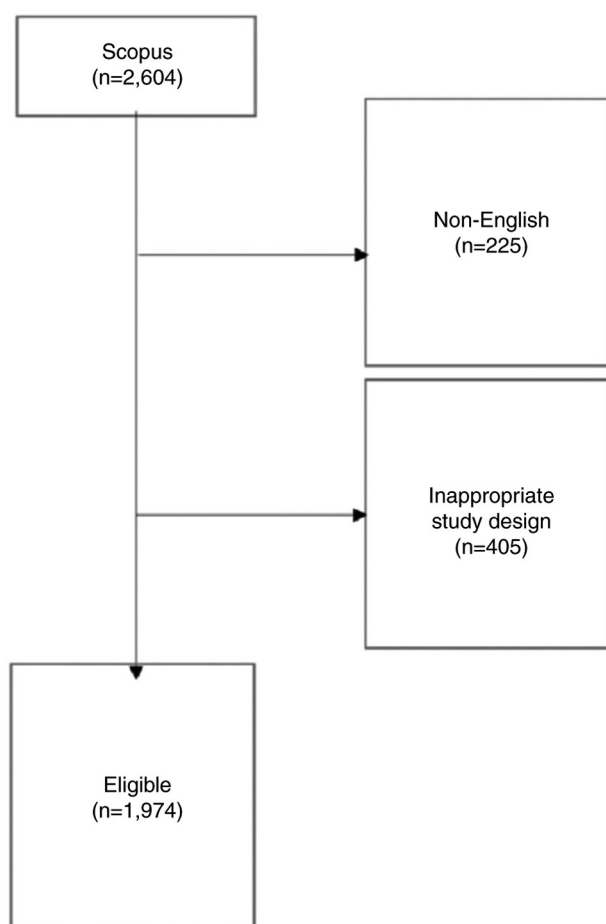

Supplement: Data extraction flow of the present study. The final analysis included 1,974 articles. [file Supplementary_Data.pdf]
